# Methods for analytical validation of novel digital clinical measures: A simulation study

**DOI:** 10.1371/journal.pone.0308190

**Published:** 2026-05-13

**Authors:** Simon Turner, Chen Chen, Rolando Acosta, Rachell Chon, Eric J. Daza, Lysbeth Floden, Joss Langford, Leif Simmatis, Berend Terluin, Benjamin Vandendriessche, Piper Fromy

**Affiliations:** 1 Digital Medicine Society (DiMe), Boston, Massachusetts, United States of America; 2 Verily Life Sciences, South San Francisco, California, United States of America; 3 Regeneron Pharmaceuticals Inc., Tarrytown, New York, United States of America; 4 College of Osteopathic Medicine, Rocky Vista University, Utah, United States of America; 5 Stats-of-1, Menlo Park, California, United States of America; 6 Evinova, Waltham, Massachusetts, United States of America; 7 Activinsights Ltd., Kimbolton, United Kingdom; 8 College of Life and Environmental Sciences, University of Exeter, Exeter, United Kingdom; 9 Department of Speech-Language Pathology, Temerty Faculty of Medicine, University of Toronto, Toronto, Ontario, Canada; 10 Department of General Practice, Amsterdam UMC, location Vrije Universiteit Amsterdam, Amsterdam, The Netherlands; 11 Amsterdam Public Health research institute, Amsterdam, The Netherlands; 12 Department of Electrical, Computer, and Systems Engineering, Case Western Reserve University, Cleveland, Ohio, United States of America; Sri Akilandeswari Women’s College, INDIA

## Abstract

Analytical validation is a crucial step in the evaluation of algorithms that process data from sensor-based digital health technologies (sDHTs). Analytical validation of novel digital measures can be complicated when reference measures with directly comparable units are not available. To address this, we conducted a simulation study. Data was simulated assuming a latent physical ability trait, indirectly accessed through an sDHT-derived target measure collecting step count data, and the items of a clinical outcome assessment (COA) measuring self-reported physical activity. We quantified the ability of two methods to assess the latent relationship between reference and target measures: the Pearson Correlation Coefficient (PCC) and factor correlations from a two-factor confirmatory factor analysis (CFA) model. Additionally, three multiple linear regression models were used to evaluate if multiple COA reference measures can more completely represent a target measure of interest. Our findings show that PCC was more stable, easier to compute, and relatively robust with respect to violations of parametric assumptions than CFA, particularly with small sample sizes. However, CFA was less biased than PCC in all scenarios investigated. We demonstrate that using both PCC and CFA generates more confidence in the results of a target and reference measure comparison. Finally, regression results suggest that incorporating multiple reference measures with more frequent collection time points can provide a more complete presentation of the sDHT’s analytical validity. Novel digital measures are being developed at an accelerating pace and promise to revolutionize patient care and medical product development. Our findings provide investigators with crucial information for choosing appropriate methods to perform rigorous analytical validation of these novel measures, including an open-access simulation toolkit.

## Introduction

For a sensor-based digital health technology (sDHT) to aid scientific and clinical decision-making, its sensors must be verified against a technical specification, algorithms analytically validated, usability validated, and clinical validation conducted to ensure relevance in specific contexts, as outlined in the V3 + framework by the Digital Medicine Society (DiMe) [1,2].

Analytical validation confirms that an sDHT algorithm accurately captures physiological or behavioral measures against a reference standard. How to identify a fit-for-purpose reference measure is outlined in the framework developed by Bakker et al. [3]). When no reference measures exist with directly comparable units, clinician-reported outcomes (ClinROs) and patient-reported outcomes (PROs) are often used [4,5], particularly in challenging therapeutic areas [6,7] such as neurodegenerative diseases [8], mental health, and cardiometabolics [9,10].

However, these types of reported episodic reference measures may not correlate well with intensive longitudinal data from sDHTs [11], complicating analytical validation. They typically do not directly assess the desired concept or construct and have inherent measurement errors that make it challenging to compare them directly to a sDHT-derived measure. Directly evaluating the correspondence between the target and reference measures is often infeasible, and may not be needed.

For instance, the Movement Disorder Society-Unified Parkinson’s Disease Rating Scale (MDS-UPDRS) [12] is a common reference for Parkinson’s disease but is known to be subjective with high within-subject variability and low test-retest reliability [13]. Comparisons between sDHT measures and MDS-UPDRS often yield poor results, which does not necessarily disprove the validity of the sDHT-derived measure.

The focus has shifted from replicating MDS-UPDRS components to developing fit-for-purpose sDHT measures that objectively assess similar concepts. This highlights the importance of convergent validity [14], which examines relationships between sDHT outputs and reference measures without directly comparable units, assuming the existence of an underlying latent trait. Statistical methods like correlation, regression, and confirmatory factor analysis (CFA) can estimate these relationships.

Our work explores the statistical properties of Pearson correlation coefficients (PCC) and confirmatory factor analysis (CFA) in evaluating convergent validity. We also investigate the validity of a given target measure by evaluating how multiple reference measures can improve an analytical validation strategy. These results should be used to inform statistical analysis planning when conducting analytical validation with a reference measure without directly comparable units. Additionally, the simulation approach provides a way to test an analytical validation approach *in silico* before investing additional resources.

## Methods

We evaluated statistical methods for analytically validating a digital measure (target) against reference measures of clinical outcome assessments (COAs). We simulated total daily step count data from a fictive sDHT as a measure of physical activity. The associated latent trait is physical ability which fluctuates daily. Additionally, we simulated COAs that measure patient or clinician-reported physical activity.

### Software

All simulations and analyses were conducted in R version 4.3.3 (R Core Team, 2021) on a Windows 11 operating system.

### Data generation mechanism (DGM)

**Fig 1:**
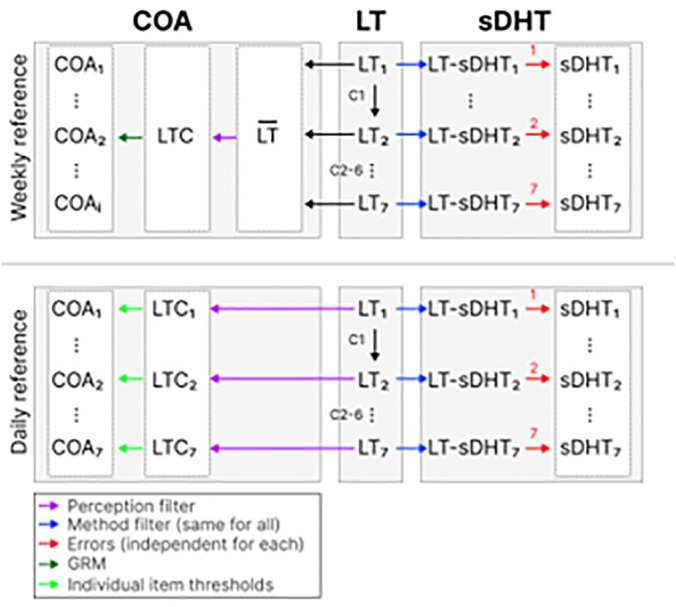
A diagram depicting the DGM used in this study, illustrating the relationship between the latent and observable variables for the target measure (sDHT-based) and the different reference measures (COA-based). C{1-6} = fluctuation factors 1-6; COA = clinical outcome assessment; GRM = graded response model; LT = latent trait; LTC = latent trait for the COA; LT-sDHT = latent trait for the sDHT.

We simulated seven days of sDHT data (i.e., the target measure), and data from multiple COAs (i.e., the reference measures). The seven-day period was chosen to balance the need for sufficient data collection while minimizing participant burden and potential dropout rates in real-world studies. Fig 1 depicts a summary of the DGM we used (Table 1).

**Table 1:** Notation used in the DGM.

Notation	Description
$\theta_{ij}$	The value of the latent variable for individual j on day i of the study
$\overset{\prime }{\underset{ij}{\theta }}$	The value of the “method-filtered” latent variable for individual j on day i of the study
$\lambda_{ij}$	The mean of the Poisson distribution used to generate individual j’s target measure data from day i
$\lambda_{O}$	The base rate for the target measure (i.e., the mean of the target measure for a latent variable value of zero)
$\lambda_{effect}$	The effect of the latent variable on the daily mean of the target measure
$\epsilon_{ij}$	The measurement error of the sDHT
${\sigma_{ij}}^{\text{2}}$	The magnitude of the sDHT measurement error (MEM)
$\theta_{j}$	The mean value of the latent trait over the seven days for individual j
$\underset{―}{{\theta_{j}}^{P}}$	Individual j’s “perception-filtered” latent trait for the weekly PRO
$DHT_{ij}$	The observed count data for individual j on day i
$wPRO_{j}$	Rescaled total score from the weekly PRO for individual j
${\theta_{ij}}^{P}$	Individual j’s perceived latent trait value for the daily PRO on day i

### Simulating the latent variables

The data generation mechanism simulated latent variables from a standard Normal distribution. The latent variable for each subsequent day was simulated by adding a fluctuation factor to the latent variable from the previous day, and then rescaling to give a sample variance of 1.0 for the data across all individuals on that day. The fluctuation factor was taken from a Normal variable with mean 0 and standard deviation (SD) 0.30. This SD value represents a moderate daily variability in physical ability, based on the physical activity patterns referenced in Fig 1.

### Simulating the target measure data

We chose to use the stimulation of seven daily counts for each individual based on contexts-of-use and population data [15–17]. Data were to be assessed on consecutive days; effects of weekdays and weekends were not specified.

To mimic the imperfect ability of the target measure to observe the latent trait, Gaussian noise with mean 0 and SD 0.5 was added to $\theta_{ij}$ to obtain $\theta_{ij}\prime$ for individual j on day i. This “method filter” did not vary between days.

To simulate the count data, a random value was drawn from a Poisson distribution with mean $\lambda_{ij}$, where $\lambda_{ij}$ was calculated as:


$$\begin{array}{c} \lambda_{ij}\;=\;\lambda_{O}\;+\;\lambda_{effect}.{\theta_{ij}}^{\prime }\;+\;\epsilon_{ij} \end{array}$$

Here, $\;\lambda_{O}$ is the hypothesized mean of the target measure for a latent variable value of zero (i.e., an individual from the population with mean physical ability). This term is independent of an individual’s latent ability. In this study, $\lambda_{O}$ = 10,000. The $\lambda_{effect}$ term represents the proportional effect of an individual’s latent physical ability on their expected target measure count, and scales an individual’s “filtered” latent trait. We set $\lambda_{effect}$ = 1,250 (i.e., the effect of latent ability on step count). The final term ($\epsilon_{ij}$) represents measurement error, and is Normally distributed with mean 0 and SD ${\sigma_{ij}}^{\text{2}}$. This term is varied as a simulation parameter. Each Poisson mean was constrained as $\lambda_{ij}>\text{0}$. The observed count data for individual j on day i is denoted as $DHT_{ij}.$ The values were chosen to reflect a correlation matrix representative of real-world physical activity measurements [18], considering typical step count ranges and variability observed in population-based studies [15–17].

### Simulating the reference measure data

#### Primary reference measure: Weekly PRO.

The primary reference measure for the study is a weekly PRO, a four-response, twelve-item patient-reported outcome administered at the end of the seven-day period. Since each individual is assumed to recall information across the entire study period when responding to the PRO, their daily latent variable values influence their answers. To model this, we used the mean of each individual’s latent traits over the seven simulated days, denoted as $\underset{―}{\theta_{j}}$, as the starting point.

We assumed individuals have an imperfect ability to perceive their $\underset{―}{\theta_{j}}$ value when recalling their activities during the previous seven days. To simulate this, we added random noise generated from a Normal distribution with mean 0 and SD 1.0. $\underset{―}{{\theta_{j}}^{P}}$ denotes the “perception-filtered” latent trait for each individual j.

Each individual’s $\underset{―}{{\theta_{j}}^{P}}$ value was then used to simulate their responses to the twelve-item PRO with four-response options. The item scores were simulated using an item response theory (IRT) graded response model (GRM). The difficulty threshold parameters took the following fixed values across each individual:


$$\begin{array}{c} \beta_{\text{2}}\;=\;(-\text{1},-\text{0}.\text{8},-\text{0}.\text{8},-\text{0}.\text{4},-\text{0}.\text{4},\text{0},\text{0},\text{0}.\text{4},\text{0}.\text{4},\text{0}.\text{8},\text{0}.\text{8},\text{1}{)}^{T} \end{array}$$

$$\begin{array}{c} \beta_{\text{1}}\;=\;\beta_{\text{2}}\;-\text{1} \end{array}$$

$$\begin{array}{c} \beta_{\text{3}}\;=\;\beta_{\text{2}}\;+\;\text{1} \end{array}$$

The discrimination parameter α was set to a fixed value across each individual, chosen to result in an approximate reliability of 0.8 for the simulated PRO. A total score was then derived as the sum of the item scores, rescaled to a 0–100 scale. These total scores are the final reference measure data that were compared to the target measure data, and are denoted by $wPRO_{j}$ for individual j.

### Secondary reference measures: Weekly ClinRO and daily PRO

The secondary aim of this study investigates whether introducing multiple reference measures could provide more information for assessing the convergent validity of a digital measure. We simulated two additional types of clinical outcome assessments: (1) weekly ClinRO: A 7-item questionnaire with 5 response options; and (2) daily PRO: A single-item measure with 5 response options.

The weekly ClinRO was simulated in a similar way to the weekly PRO. We simulated an individual’s imperfect recall over the seven days by applying a perception filter to $\underset{―}{\theta_{j}}$ and adding random noise generated from a Normal distribution with mean 0 and SD 1.0. We then used an IRT GRM to simulate each individual’s responses to the seven items on the five-point scale. The difficulty threshold parameters took the following fixed values across each individual:


$$\begin{array}{c} \beta_{\text{1}}\;=\;\beta \;-\;\text{1}\;,\;\beta_{\text{2}}\;=\;\beta \;-\;\frac{\text{1}}{\text{3}}\;,\;\beta_{\text{3}}\;=\;\beta \;+\;\frac{\text{1}}{\text{3}}\;,\;\beta_{\text{4}}\;=\;\beta \;+\;\text{1} \end{array}$$

Here,


$$\begin{array}{c} \beta \;=\;(-0.75,-0.5,-0.25,\;0,\;0.25,\;0.5,\;0.75{)}^{T} \end{array}$$

The discrimination parameter α was set to a fixed value across each individual, chosen to result in an approximate reliability of 0.7 for the simulated ClinRO. A total score was calculated as the sum of the item scores, rescaled to a 0–100 scale. These rescaled total scores were the variables analyzed in our study.

The single-item daily PRO was simulated using the approach in Griffiths et al [19]. The response of an individual j to a single item (on a five-point [0–4] scale) on day i depends on $\theta_{ij}$.

As before, we added random noise generated from a Normal distribution with mean 0 and SD 0.5 to mimic the imperfect ability of an individual to perceive their $\theta_{ij}$ value. This “perceived latent trait” value is denoted ${\theta_{ij}}^{P}$, and is assumed to exactly determine (i.e., without noise) the response of individual j on day i.

We assumed the thresholds at which an individual gives a particular response were −1.5, −0.5, 0.5, and 1.5. For example, a perceived latent trait value of −1.6 should on average result in a score of 0; a perceived latent trait value of 0.8 should on average result in a score of 3.

Finally, we assume that these score threshold values vary slightly based on an individual’s own perception. What one individual may consider to be “vigorous” physical activity, another may consider “moderate”. Therefore, each individual’s score thresholds were generated from Normal distributions with means −1.5, −0.5, 0.5 and 1.5, each with SD 0.075. An individual’s ${\theta_{ij}}^{P}$value was then compared against their score thresholds to determine their daily PRO score on day i, and this process was repeated for each of the seven days in the study.

### Statistical methods

We used PCC and CFA to assess the relationship between the sDHT measure and the weekly PRO. Of note, we assumed Normal distributions for many of our variables. This may not apply to all clinical variables in real-world scenarios.

First, the true relationship between the target measure and the reference measure was calculated. This was done by computing $\rho (\underset{―}{\overset{\prime }{\underset{j}{\theta }}}\;,\;\underset{―}{{\theta_{j}}^{P}})$, the linear correlation between the mean of the sDHT “method-filtered” latent values and the “perception-filtered” mean of the latent values.

**Fig 2:**
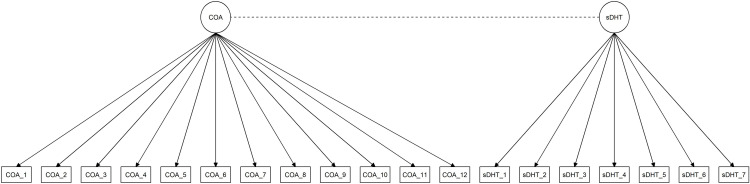
Path diagram for the CFA model used in investigating the primary aim of the study.

Two estimates of the true relationship were computed. First, the PCC between the weekly mean of the observed target measure values ($\underset{―}{DHT_{ij}})$ and the observed reference measure values ($wPRO_{j}$) was computed. Second, a two-factor CFA model with correlated factors was created, and the model-implied correlation coefficient of the factors was obtained. To construct the CFA model, each item from the reference measure was loaded as an indicator onto a “reference measure factor”, and each day of the target measure data was loaded as an indicator onto a separate “sDHT” factor. The standardized value of the factor covariance was computed, and these were the factor correlation variables analyzed in our study. Fig 2 depicts the path diagram for the two-factor CFA model.

For the secondary aim of the study, simple linear regression (SLR) and multiple linear regression (MLR) were used to assess the impact of using more than one reference measure to conduct analytical validation.

For each regression model, $\underset{―}{DHT_{ij}}$ was used as the dependent variable. Ordinary least squares was used for the SLR model, with $wPRO_{j}$ as the predictor variable. MLR was employed in three different ways: First, by introducing the weekly ClinRO as a predictor variable alongside the weekly PRO (the “weekly COAs model”); second, by introducing the mean of the daily PRO values as a third predictor variable alongside the weekly COAs (the “daily means” model); and lastly, by instead introducing the individual daily PRO scores as seven distinct predictor variables alongside the weekly COAs (the “daily distinct” model).

For each linear regression model used, a coefficient of determination was computed: R^2^ statistic in the SLR case and adjusted R^2^ in the MLR case, calculated as $\underset{―}{R^{\text{2}}}=\;\text{1}\;-\;(\text{1}-R^{\text{2}})\frac{n-\text{1}}{n-p-\text{1}}$, where n is the number of observations and p is the number of reference measures.

### Parameters of the simulation

The simulation varied the number of included COA measures and other parameters to assess their impact on the performance of the statistical methods investigated. A full factorial design was used to test the following parameters:

1. Sample size (N = 35, 100, 200, 1000)
2. Number of repeated assessments of the target measure included in the analysis (RA = 1, 3, 5, 7)
3. Missing data rate (MDR = 0, 0.10, 0.25, 0.40)
4. Missing data mechanism (No missing data, missing completely at random (MCAR), missing not at random (MNAR)) [20,21]
5. sDHT-measurement error magnitude (MEM):${{(\sigma }_{ij}}^{\text{2}}\;=\;\frac{\text{1}}{\text{2}}\lambda_{effect}\;,\;\lambda_{effect}\;,\;{\frac{\text{3}}{\text{2}}\lambda }_{effect}\;,\;\text{2}\lambda_{effect})$

For a more detailed explanation of the missingness mechanisms employed, and the implementation of fewer repeated assessments of the target measure, in the simulation, please see the Supplementary Materials.

### Performance measures

#### Primary aim of the study.

To assess the ability of PCC and CFA factor correlation to observe the underlying relationship between the target and primary reference measure, the empirical bias of both methods was computed. The empirical bias of a method, defined in terms of statistical estimators, is defined as the difference between 1) the true relationship between the target measure and the reference measure, and 2) the sample-based estimate of this true relationship from that method. The empirical bias for PCC is the difference between $\rho (\underset{―}{\overset{\prime }{\underset{ij}{\theta }}}\;,\;\underset{―}{{\theta_{j}}^{P}})$ and $\rho (\underset{―}{DHT_{ij}}\;,\;wPRO_{j})$. For CFA, the empirical bias is the difference between $\rho (\underset{―}{\overset{\prime }{\underset{ij}{\theta }}}\;,\;\underset{―}{{\theta_{j}}^{P}})$ and the factor correlation estimate.

To assess the precision of the above methods, the empirical standard error (empSE) of the PCC and the CFA factor correlation were computed for each simulation condition. EmpSE is defined as the square root of the variance of each method’s correlation estimates.

In addition, four model fit statistics were calculated for the CFA model to evaluate the goodness of fit between the proposed model and the observed data. The Comparative Fit Index (CFI), Tucker-Lewis Index (TLI), Root Mean Square Error of Approximation (RMSEA), and Standardized Root Mean Square Residual (SRMR) were used as fit statistics. The rate for which the model fit statistics suggested an acceptable model fit are reported for each simulation condition. Interpretation of the fit statistics was guided by the following thresholds [22,23]: (1) CFI and TLI acceptable fit: values ≥ 0.90; and (2) RMSEA and SRMR acceptable fit: values < 0.08. The convergence, or failure rates ($\frac{number\;of\;non-convergences}{total\;number\;of\;attempts}$), for the CFA model are reported for each simulation condition.

### Secondary aim of the study

To assess the ability of the MLR models to recover more information about the underlying target and reference measure relationship than the SLR model, the change in information added, denoted $\Delta R^{2}$, was calculated for each MLR model. $\Delta R^{2}$ is defined as the difference between the MLR adjusted R^2^ estimate and the SLR R^2^ estimate; in other words, $\Delta R^{2}\;=\;\underset{―}{R^{2}}-\;R^{2}$. To assess the precision of each regression method, for each simulation condition, the empSE of the SLR model and each MLR model were calculated. Finally, any failures that occurred in each of the regression models are reported.

### Number of simulation repetitions and Monte Carlo standard error

The number of simulation repetitions (*n*_sim_) was chosen to ensure that the Monte Carlo Standard Error (MCSE) of the empSE for each statistical method was less than 0.025. To achieve this, *n*_sim_ must satisfy the inequality $n_{sim}\;\geq \;\text{1}\;+\;\text{8}\text{0}\text{0}{{\;S}_{\hat{R}}}^{\text{2}}$, where ${{\;S}_{\hat{R}}}^{\text{2}}$ is the sample variance of the estimates [24,25]. Based on a test simulation, n_sim_ = 500 is sufficient to achieve the desired MCSE. The MCSEs of each model are reported for each simulation condition.

## Results

### Primary aim of the study

Table 2 depicts a summary of the mean empirical bias and mean empSE for the PCC and CFA methods under the influence of different simulation conditions, as well as an overall summary across all conditions.

**Table 2:** Mean (SD) of the empirical bias and EmpSE for each method.

	Mean (SD) Empirical Bias		Mean (SD) Empirical SE	
	*Pearson R of raw measures*	*CFA factor correlation*	*Pearson R of raw measures*	*CFA factor correlation*
**Overall**	−0.160 (0.107)	0.012 (0.111)	0.076 (0.046)	0.100 (0.071)
By Sample Size				
35	−0.159 (0.145)	0.024 (0.189)	0.140 (0.029)	0.193 (0.071)
100	−0.161 (0.104)	0.014 (0.093)	0.081 (0.017)	0.103 (0.030)
200	−0.161 (0.089)	0.008 (0.065)	0.057 (0.011)	0.072 (0.021)
1000	−0.161 (0.077)	0.002 (0.029)	0.025 (0.005)	0.032 (0.009)
By MEM				
$\frac{\text{1}}{\text{2}}\lambda_{effect}$	−0.079 (0.061)	0.013 (0.066)	0.068 (0.040)	0.073 (0.043)
$\lambda_{effect}$	−0.132 (0.080)	0.018 (0.08)	0.073 (0.044)	0.083 (0.048)
${\frac{\text{3}}{\text{2}}\lambda }_{effect}$	−0.190 (0.097)	0.016 (0.112)	0.079 (0.047)	0.104 (0.068)
${\text{2}\lambda }_{effect}$	−0.241 (0.108)	0.001 (0.161)	0.083 (0.049)	0.139 (0.097)
By number of sDHT assessments				
1	−0.217 (0.132)	N/A	0.093 (0.055)	N/A
3	−0.160 (0.101)	0.010 (0.121)	0.076 (0.044)	0.112 (0.074)
5	−0.138 (0.086)	0.012 (0.108)	0.070 (0.040)	0.097 (0.071)
7	−0.126 (0.077)	0.014 (0.102)	0.066 (0.037)	0.091 (0.069)
By missing data mechanism				
None	−0.145 (0.095)	0.016 (0.081)	0.067 (0.038)	0.082 (0.053)
MCAR	−0.159 (0.111)	0.017 (0.116)	0.082 (0.049)	0.106 (0.074)
MNAR	−0.167 (0.106)	0.006 (0.114)	0.073 (0.043)	0.100 (0.074)
By missing data rate				
0	−0.145 (0.095)	0.016 (0.081)	0.067 (0.038)	0.082 (0.053)
0.10	−0.151 (0.099)	0.016 (0.090)	0.070 (0.041)	0.089 (0.058)
0.25	−0.162 (0.107)	0.013 (0.108)	0.076 (0.045)	0.099 (0.068)
0.40	−0.177 (0.118)	0.004 (0.141)	0.086 (0.052)	0.121 (0.089)

### Empirical Bias

Overall, PCC was negatively biased and CFA was positively biased, but CFA was the less-biased of the two methods. The same behavior was observed when splitting the bias results by any single simulation parameter. When considering the impact of any single simulation parameter on the mean empirical bias, the negative bias of PCC increased as the MEM and missing data rate increased. The choice of missing data mechanism only had a minor impact on the mean empirical bias of PCC, and the impact of sample size was negligible. The negative bias of PCC decreased as the number of repeated assessments increased.

For the CFA method, any single simulation parameter had little impact on the mean empirical bias. At the maximum MEM level, the CFA method was slightly less biased than for lower MEM values; the same behavior was observed for the maximum missing data rate. Data that were MNAR, produced a slightly smaller magnitude of the bias when compared to no missing data or MCAR data. Varying the number of repeated target measure assessments, had no discernible impact on the mean empirical bias. When increasing the sample size, there was a very slight trend for the mean empirical bias of CFA to decrease, and this was the strongest of the trends exhibited when considering a single simulation condition.

**Fig 3:**
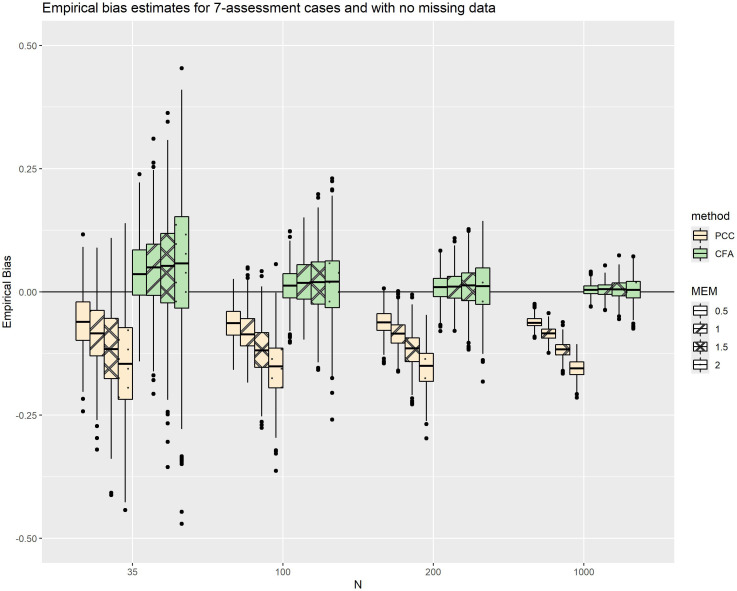
Empirical bias estimates for PCC and CFA factor correlations, as a function of increasing MEM, with no missing data and a fixed maximum number of repeated assessments.

Focusing on a specific simulation condition (no missing data and 7 repeated assessments per subject; Fig 3), sample size impacted the average trend of empirical bias less than MEM, while the dispersion of empirical bias appeared to decrease as sample size increased. We observed a pattern of increasing underestimation from PCC as MEM increased. In contrast, CFA slightly overestimated the bias, but the average trend was not greatly affected by the varying measurement error. This pattern was repeated in the 3-assessment and 5-assessment case.

### EmpSE

**Fig 4:**
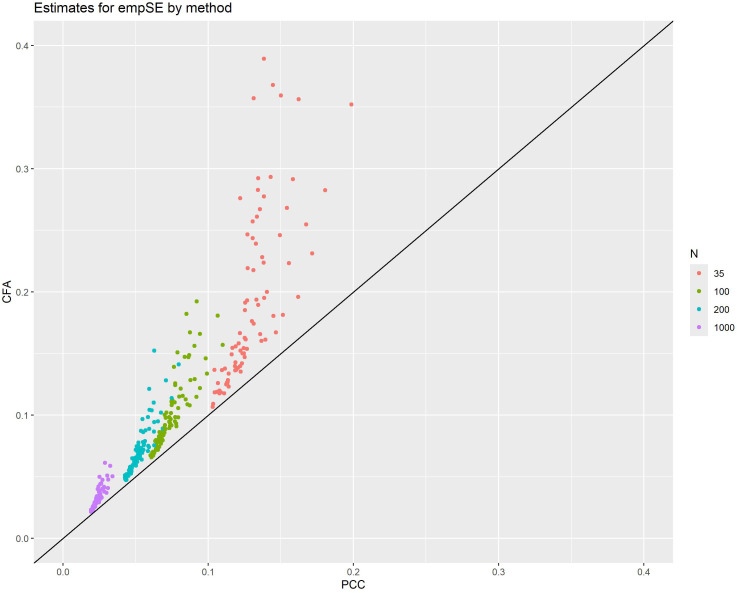
Plot of the empSEs for PCC against the empSEs for CFA factor correlation with line of identity. Colors represent the different sample sizes.

Table 2 shows that, overall, PCC had a lower mean empSE than CFA. In fact, a stronger condition is true – for every combination of simulation parameters investigated in the study, the empSE of CFA was greater than the corresponding empSE of PCC. This is also illustrated by Fig 4, which shows the empSEs of PCC plotted against the empSEs of the CFA factor correlation, colored by sample size.

Continuing with Table 2, when considering the impact of any single simulation parameter on the mean empSE, decreasing the MEM, or the missingness rate caused the mean empSE to decrease for both PCC and CFA. For both methods, the empSE was lower with no missing data compared to that for MNAR data. The empSE for MNAR data was lower than that for MCAR data. When sample size increased, the mean empSE of both methods decreased. An increase in the number of assessments corresponded with a decrease in mean empSE for both methods. These trends persisted in more granular simulation scenarios, such as the one shown in Table 3 (i.e., no missing data, seven repeated assessments).

**Table 3:** Empirical SE for each condition shown in Fig 5a.

Sample size	MEM				Method
	$\frac{1}{2}\lambda_{effect}$	$\lambda_{effect}$	${\frac{3}{2}\lambda }_{effect}$	${2\lambda }_{effect}$	
35	0.105	0.108	0.119	0.126	Pearson
	0.118	0.118	0.136	0.191	CFA
100	0.061	0.066	0.066	0.070	Pearson
	0.066	0.074	0.080	0.090	CFA
200	0.044	0.044	0.048	0.051	Pearson
	0.047	0.049	0.058	0.067	CFA
1000	0.020	0.020	0.021	0.022	Pearson
	0.022	0.023	0.026	0.029	CFA

**Fig 5:**
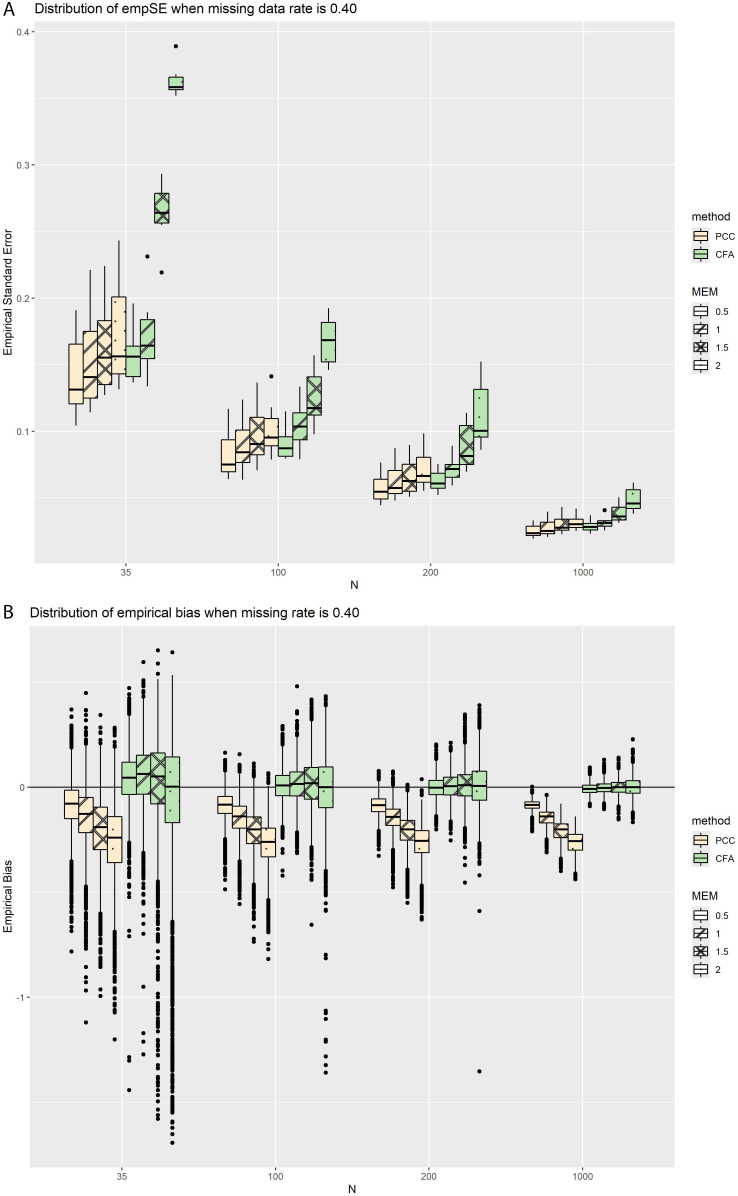
(a) EmpSEs and (b) empirical biases for both methods, as a function of increasing sample size with a fixed missing data rate of 0.40.

Fig 5a depicts the empSEs for PCC and CFA factor correlations, as a function of increasing sample size with a fixed missing data rate of 0.40; Fig 5b depicts the corresponding empirical biases. When holding the missing data rate constant and allowing sample size to vary (Fig 5a), we observed a pattern of increasing empSE with increasing MEM, which was mitigated by increasing sample size. For example, the difference between the median empSE at the highest and lowest MEM levels in the PCC condition was 0.025 for n = 35, whereas the difference between the highest and lowest MEM values was only 0.007 for n = 1000 (i.e., a 3.6-fold difference). Furthermore, the empSE of CFA was more sensitive to increasing MEM at all sample sizes. The difference between the median empSE at the highest and lowest MEM levels was 0.202 and 0.018, for the n = 35 and n = 1000 cases respectively (i.e., an 11.2-fold difference). While the empSE of PCC also grew as MEM increased, its impact was less severe across all sample sizes. Empirical biases (Fig 5b) in these scenarios did not behave differently than the overall trend (Table 2).

**Fig 6:**
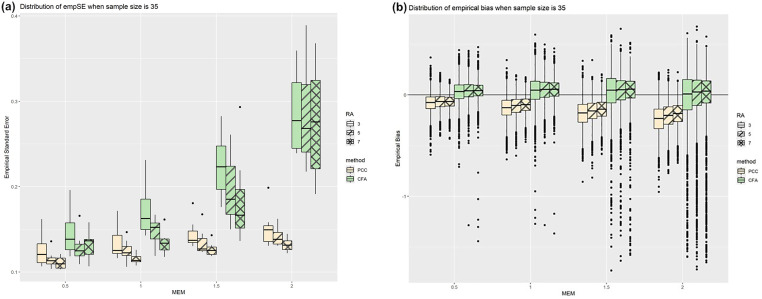
(a) EmpSEs and (b) empirical biases for both methods, as functions of MEM with a fixed sample size of 35.

When holding sample size constant (n = 35) and allowing MEM to vary, there was a trend for the empSE (Fig 6a) to perform notably worse in the 3-assessment cases than larger number of repeated assessments (5 and 7); this trend was mitigated by reducing the MEM, except that for the largest simulated MEM ($\;\text{2}\lambda_{effect}$) empSE for all repeated assessments were equally poor. While following a similar trend, PCC has a notably smaller empSE across these scenarios as compared to CFA correlations. The same pattern holds in larger sample sizes (see S2a Fig in S1 File for n = 100). Empirical biases (Fig 6b and S2b Fig in S1 File) did not differ notably from the overall trend (Table 2).

### CFA model fit statistics: rates of acceptable fit and failure rates

Table 4 depicts a summary of the rates of acceptable fit for each CFA model fit statistic and the model failure rate under the influence of different simulation conditions, as well as an overall summary across all conditions.

**Table 4:** Mean rates of acceptable fit for each model fit statistic, grouped in various ways.

	Mean (SD) rates of acceptable fit^*^				*Mean (SD) of Failure rate* ^*#*^
	*CFI*	*TLI*	*RMSEA*	*SRMR*	
**Overall**	84.8% (28.1%)	82.2% (30.6%)	45.4% (44.6%)	58.0% (45.3%)	2.67% (5.74%)
By Sample Size					
35	59.1% (36.8%)	54.5% (38.0%)	20.0% (31.8%)	<0.01% (0.10%)	10.1% (7.48%)
100	84.2% (25.7%)	80.3% (29.3%)	31.4% (40.3%)	34.7% (30.6%)	0.51% (1.56%)
200	96.0% (12.0%)	94.2% (15%)	45.3% (44.9%)	97.2% (7.6%)	0.06% (0.30%)
1000	100% (0%)	100% (0%)	84.7% (30.8%)	100% (0%)	0% (0%)
By MEM					
$\frac{\text{1}}{\text{2}}\lambda_{effect}$	99.6% (1.30%)	99.4% (2.00%)	89.9% (18.5%)	67.1% (42.8%)	1.37% (2.47%)
$\lambda_{effect}$	96% (10.0%)	94.6% (12.7%)	52.3% (41.5%)	60.3% (44.4%)	1.52% (2.8%)
${\frac{\text{3}}{\text{2}}\lambda }_{effect}$	81.8% (27.9%)	78.2% (30.8%)	26.7% (40.2%)	54.3% (46.1%)	2.72% (5.29%)
${\text{2}\lambda }_{effect}$	61.9% (37.7%)	56.7% (39.1%)	12.6% (28.9%)	50.2% (46.9%)	5.07% (9.07%)
By number of sDHT assessments					
1	N/A	N/A	N/A	N/A	N/A
3	88.9% (21.5%)	86.1% (24.6%)	55.3% (43.3%)	60.2% (44.3%)	3.83% (7.86%)
5	84.3% (28.7%)	81.6% (31.4%)	44.3% (44.6%)	57.5% (45.7%)	2.17% (4.47%)
7	81.3% (32.7%)	79% (34.7%)	36.5% (44.2%)	56.2% 46.2%)	2.01% (3.97%)
By missing data mechanism					
None	91.4% (21.1%)	89.5% (23.8%)	56.0% (45.3%)	65.0% (43.6%)	1.77% (3.60%)
MCAR	84.7% (27.7%)	82.0% (30.2%)	43.9% (44.3%)	56.4% (45.6%)	2.93% (6.29%)
MNAR	82.8% (30.3%)	80.1% (32.7%)	43.3% (44.4%)	57.1% (45.7%)	2.71% (5.76%)
By missing data rate					
0	91.4% (21.1%)	89.5% (23.8%)	56.0% (45.3%)	65.0% (43.6%)	1.77% (3.60%)
0.10	89.1% (24.0%)	87.1% (26.5%)	51.7% (45.3%)	62.3% (44.3%)	2.08% (4.22%)
0.25	84.7% (28.0%)	82.0% (30.4%)	43.9% (44.4%)	57.3% (45.7%)	2.47% (5.21%)
0.40	77.3% (33.3%)	74.0% (35.6%)	35.1% (41.9%)	50.9% (46.3%)	3.91% (7.92%)

*acceptable fit is defined as those simulation models with a CFI or TLI value of at least 0.9, and a RMSEA or SRMR value of less than 0.08; a failed model is one that resulted in an error output from the simulation code or returned a factor correlation not in the interval [−1,1].

Across all conditions combined, fit statistics CFI and TLI were generally within acceptable bounds (84.8% and 82.2% respectively), and SRMR were generally acceptable but lower (58.0%). RMSEA were the least consistently acceptable, with only 45.4% meeting the criterion.

When considering the impact of varying any single simulation condition, increasing the parameters that control information quality (MEM and missing data rate) reduced the mean rate of acceptable fit across all fit statistics. MEM had a particularly large impact on RMSEA, decreasing the mean rate of acceptable fit by 67.3% as MEM increased from the minimum to the maximum value. The equivalent decrease in mean acceptable fit for SRMR was 16.9%, 37.7% for CFI and 42.7% for TLI. Varying the missing data mechanism had a much smaller impact on the mean acceptable rate for any of the fit statistics.

Increasing the sample size increased the mean acceptable fit rate for each statistic, especially for RMSEA and SRMR. For n = 1000, CFI, TFI and SRMR always indicated an acceptable model fit, whereas RMSEA had an acceptable fit rate 84.7%. For n = 35, almost none of the models were an acceptable fit according to SRMR, less than third were an acceptable fit according to RMSEA, and fewer than two-thirds were an acceptable fit according to CFI and TLI. Based on SRMR, almost all of the unacceptable models had a sample size of n ≤ 100.

Increasing the number of repeated assessments decreased the mean rate of acceptable fit for each fit statistic. The decrease in rate of acceptability was slight for each of CFI, TLI and SRMR, and slightly greater for RMSEA.

**Fig 7:**
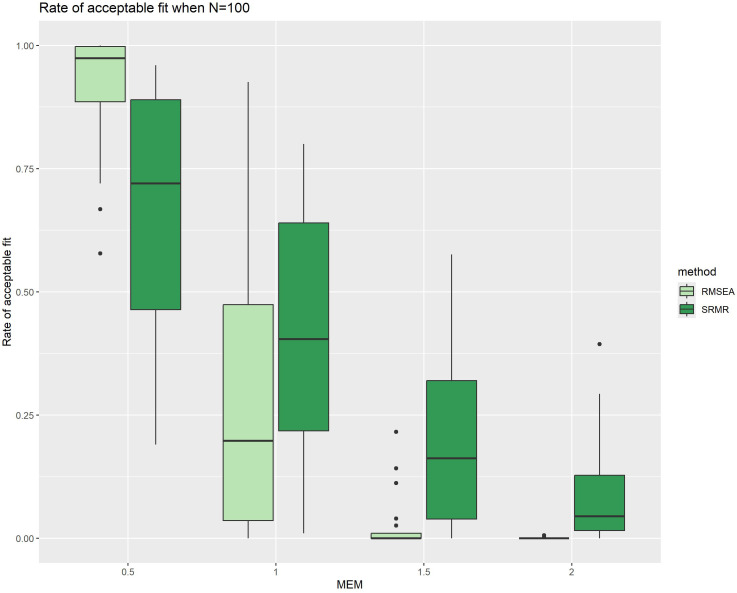
Rate of RMSEA and SRMR acceptable fit, grouped by MEM, when restricted to n = 100.

Fig 7 shows the rate of acceptable fit according to RMSEA and SRMR, restricted to n = 100 and grouped by MEM. Fig 7 shows that the model fit according to RMSEA when n = 100 was generally acceptable when MEM was at the minimum level of $\frac{\text{1}}{\text{2}}\lambda_{effect}$; the mean acceptable fit rate was 91.2% compared to 31.4% when considering all MEM conditions together for n = 100. In contrast, almost no models had an acceptable RMSEA fit when MEM was at the maximum value – the mean acceptable rate was less than 1% in these cases as opposed to the overall 31.4% rate. The SRMR acceptability rate exhibited a similar but weaker trend to RMSEA, with a mean acceptable rate of 68.4% at minimum MEM, compared to 9.0% at maximum MEM (with an overall mean SRMR acceptable fit rate of 34.7% when n = 100).

Similar but weaker patterns were seen for RMSEA and SRMR acceptable fit when grouping the n = 100 cases by both the number of repeated assessments and by missing data rate: as the grouping parameter increased, the rate of acceptable fit decreased.

In approximately 9% of the total individual simulation repetitions, the RMSEA value was exactly zero. In all of these repetitions, the CFI was exactly 1.0 and the TLI was greater than 1.0. These repetitions were not limited to any particular combination of simulation conditions, although they were more likely to occur in conditions with lower levels of information (i.e., small number of assessments and small sample size) and better levels of signal to noise (i.e., small MEM and low missing data rate).

Almost all of the failures of the CFA model to converge occurred when sample size was 35, and sample size had the largest impact on failure rate when compared with other simulation parameters in this study. Increasing the MEM and missing data rate resulted in slight increases in failure rate. When including repeated target measure assessments, increasing the number of assessments led to a reduction in failure rate.

When attempting to use a single target measure assessment in the CFA model, the model was consistently non-identifiable (i.e., multiple model specifications fit the data equally well). While factor correlations were computed in these cases, they are not been reported as they are unlikely to be meaningful.

### Secondary aim of the study

Table 5 depicts a summary of the mean change in information added ($\Delta R^{\text{2}}$) and mean empSE for each regression method (SLR, MLR with “daily means”, MLR with “daily distinct”, and MLR with “weekly COAs”) under the influence of the different simulation conditions, as well as an overall summary across all conditions.

**Table 5:** Mean (SD) of $\Delta R^{2}$ and empirical SE for each regression model grouped in various ways.

	Mean (SD)$\Delta R^{2}$			Mean (SD) Empirical SE			
	*MLR - “daily means” model*	*MLR - “daily distinct”*	*MLR - “weekly COAs” model*	*SLR*	*MLR - “daily means”” model*	*MLR - “daily distinct” model*	*MLR - “weekly COAs” model*
**Overall**	0.198 (0.104)	0.206 (0.123)	0.031 (0.050)	0.063 (0.036)	0.068 (0.043)	0.079 (0.064)	0.069 (0.041)
By Sample Size							
35	0.180 (0.146)	0.187 (0.190)	0.014 (0.079)	0.116 (0.015)	0.129 (0.030)	0.166 (0.068)	0.130 (0.020)
100	0.199 (0.098)	0.207 (0.104)	0.033 (0.043)	0.068 (0.009)	0.071 (0.013)	0.076 (0.017)	0.072 (0.010)
200	0.204 (0.084)	0.212 (0.086)	0.038 (0.031)	0.048 (0.007)	0.049 (0.009)	0.051 (0.011)	0.050 (0.007)
1000	0.208 (0.070)	0.216 (0.072)	0.042 (0.019)	0.022 (0.003)	0.022 (0.004)	0.022 (0.004)	0.022 (0.003)
By MEM							
$\frac{\text{1}}{\text{2}}\lambda_{effect}$	0.280 (0.081)	0.290 (0.092)	0.048 (0.050)	0.068 (0.038)	0.059 (0.038)	0.065 (0.05)	0.071 (0.043)
$\lambda_{effect}$	0.221 (0.087)	0.229 (0.104)	0.036 (0.049)	0.066 (0.037)	0.067 (0.043)	0.077 (0.060)	0.070 (0.042)
${\frac{\text{3}}{\text{2}}\lambda }_{effect}$	0.167 (0.089)	0.173 (0.113)	0.025 (0.048)	0.062 (0.035)	0.072 (0.045)	0.085 (0.069)	0.068 (0.041)
${\text{2}\lambda }_{effect}$	0.125 (0.087)	0.130 (0.117)	0.016 (0.045)	0.058 (0.033)	0.072 (0.045)	0.088 (0.075)	0.065 (0.040)
By number of sDHT assessments							
1	0.154 (0.117)	0.156 (0.152)	0.014 (0.053)	0.063 (0.038)	0.079 (0.052)	0.100 (0.090)	0.072 (0.047)
3	0.186 (0.098)	0.209 (0.118)	0.031 (0.049)	0.064 (0.037)	0.069 (0.043)	0.079 (0.060)	0.070 (0.042)
5	0.219 (0.092)	0.225 (0.102)	0.039 (0.047)	0.064 (0.035)	0.063 (0.038)	0.070 (0.048)	0.067 (0.039)
7	0.233 (0.088)	0.233 (0.095)	0.042 (0.045)	0.062 (0.034)	0.059 (0.035)	0.065 (0.043)	0.065 (0.037)
By missing data mechanism							
None	0.215 (0.096)	0.223 (0.104)	0.037 (0.045)	0.058 (0.032)	0.058 (0.035)	0.064 (0.043)	0.062 (0.036)
MCAR	0.197 (0.108)	0.205 (0.135)	0.030 (0.054)	0.068 (0.038)	0.074 (0.048)	0.089 (0.078)	0.074 (0.045)
MNAR	0.193 (0.102)	0.200 (0.114)	0.031 (0.046)	0.06 (0.034)	0.064 (0.039)	0.073 (0.053)	0.065 (0.038)
By missing data rate							
0	0.215 (0.096)	0.223 (0.104)	0.037 (0.045)	0.058 (0.032)	0.058 (0.035)	0.064 (0.043)	0.062 (0.036)
0.10	0.208 (0.099)	0.216 (0.108)	0.035 (0.046)	0.06 (0.034)	0.062 (0.037)	0.069 (0.047)	0.064 (0.037)
0.25	0.196 (0.103)	0.204 (0.118)	0.031 (0.049)	0.064 (0.036)	0.068 (0.042)	0.078 (0.058)	0.069 (0.041)
0.40	0.182 (0.111)	0.188 (0.145)	0.026 (0.055)	0.068 (0.039)	0.078 (0.051)	0.096 (0.088)	0.076 (0.048)

### Change in information added, $\Delta R^{2}$

Both MLR methods that included the daily PRO data performed well, recovering more information than the SLR model in the vast majority of cases (96.9% for the daily PRO means model, and 95.9% for the individual daily PRO days model). The MLR method including only the weekly COAs performed less well but was still generally better than the SLR model, adding more information than the SLR method in 77.8% of cases.

When considering the mean $\Delta R^{2}$, the MLR methods performed similarly well to each other, with a slight increase in mean $\Delta R^{2}$ for the “daily distinct” model as compared to the “daily means” model (0.206 and 0.198, respectively) The “weekly COAs” model performed much worse, with a mean $\Delta R^{2}$ of 0.031. This pattern was repeated when considering the results grouped by any single simulation parameter.

When grouping the $\Delta R^{2}$ results by any single simulation parameter, the largest impacts on $\Delta R^{2}$ were observed for MEM and the number of repeated assessments. In general, increasing the sample size and number of assessments led to an increase in $\Delta R^{2}$ for all models. Moreover, reducing the MEM and missing data rate also increased $\Delta R^{2}$ for all models. Sample size had a notable impact on the variability of $\Delta R^{2}$ under the daily distinct model, with an SD of 0.190 at n = 35 compared to 0.072 at n = 1000. Values of $\Delta R^{2}$ from simulations with a sample size of 35 exhibited the greatest variability of all models across all conditions.

When investigating simulations with negative $\Delta R^{2}$ values (3.1% of all values for the means model, 4.1% of all values for the daily distinct model, and 22.2% of all values for the weekly COAs model), sample size had the greatest impact out of any single simulation parameter. When sample size was 35, over half of the $\Delta R^{2}$ values for the weekly COAs model were negative, decreasing to around 25% simulations at n = 100, then around 10% at n = 200, and almost no simulation at n = 1000 produced a negative $\Delta R^{2}$ for this model. This pattern was repeated for the other two models; between 10% and 15% of simulations at n = 35 returned a negative $\Delta R^{2}$ for the means and daily distinct models, respectively, which reduced to almost zero for larger sample sizes.

There were a limited number of simulations where the MLR $\;\underset{―}{R^{2}}$values were negative; the majority of these negative values occurred for n = 35.

### EmpSE

Overall, the empSE was similar between all MLR and SLR methods. The SLR method had a slightly smaller mean empSE, with the “daily means” model and “weekly COA” models having slightly larger but broadly similar means, and the “daily distinct” model having the largest mean empSE among all four methods. When considering any single simulation parameter, varying the sample size resulted in the strongest impact on empSE, with an increase in sample size tending to result in a decrease in empSE for each regression method.

This trend was less clear when varying the number of repeated assessments. For MLR methods there was a slight trend of decrease in mean empSE as the number of assessments increased, but for the SLR method, varying the number of assessments had broadly no impact. Introducing missing data via MNAR tended to produce a slightly reduced empSE in each method as compared to MCAR, and increasing the missing data rate tended to slightly increase empSE for each method. When allowing just the MEM to vary, a more complicated picture emerged. For both models that included the daily PRO, empSE exhibited a slight tendency to increase as MEM increased. However, for methods only including weekly COAs (i.e., the SLR and the MLR “Weekly COAs model) this was reversed, and the empSE exhibited a slight tendency to decrease as MEM increased.

### Regression model failure rates

We observed a limited number of failures when the $\;\underset{―}{R^{2}}$estimate returned as NaN in the “daily distinct” MLR model, all of which occurred when sample size was 35 and missing data rate was at its maximum of 0.40 under the MCAR mechanism. Table 6 lists the specific simulation parameter combinations that led to failures, along with their failure rates.

**Table 6:** Conditions where the “day-by-day” model produced failures, along with the failure rate.

N	MEM	Number of sDHT assessments	Missing data mechanism	Missing data rate	Failure rate
35	0.5	1	MCAR	0.4	1.85%
35	1	1	MCAR	0.4	2.24%
35	1.5	1	MCAR	0.4	1.69%
35	2	1	MCAR	0.4	2.57%
35	1.5	3	MCAR	0.4	0.18%

There were a small number of $\;\underset{―}{R^{2}}$estimates produced from the daily distinct model which strongly underperformed the SLR $R^{2}$; these estimates tended to arise in the same simulation conditions where model failures were reported. The SLR model, the “daily means” MLR model, and the “weekly COAs model exhibited no failures during the simulation.

### Monte Carlo standard errors of the empSE

All MCSE values across each of the simulation conditions were smaller than the acceptability criteria of 0.025, for each of the six statistical methods investigated in this work. Table 7 shows the mean MCSE of the empSE for each method, and we observed that these values are all comfortably below the acceptability threshold.

**Table 7:** MCSEs of the mean empSEs for each statistical method investigated.

Performance Measure	Statistical Method					
	*PCC*	*CFA*	*SLR*	*MLR – daily means*	*MLR – daily distinct*	*MLR – weekly COAs*
Mean EmpSE	0.0024	0.0032	0.0020	0.0021	0.0025	0.0022

## Discussion

### Summary of results and how they help in practice

Our simulation study highlights the challenges in validating novel digital endpoints against reference measures with non directly comparable units. While sDHTs offer the potential for increasingly more objective and continuous methods of measurement, rigorous analytical validation is critical to support the continued development and implementation of such novel digital clinical measures.

This simulation study investigated the convergent validity of an sDHT-derived measure with reported reference measures. PCC and CFA factor correlations were used. The study design and parameter choices to simulate these measures were informed by real-world data and observations, including missing data mechanisms.

Our analysis found that Pearson correlation was more stable, easier to compute, and relatively robust to violations of parametric assumptions [26], making it a recommended choice for small sample size studies. However, PCC tends to underestimate the true correlation, and this underestimation becomes more pronounced as the degree of measurement error increases.

CFA factor correlation has gained popularity as its approach to extracting latent information can better mitigate the statistical impacts of measurement error on estimation. Because it is a more complex model, it requires a well-sized sample (n > 100) and frequent repeated assessments from the sDHT. We deemed the CFA model unsuitable for a single assessment scenario due to model non-identifiability. Additionally, model fitting evaluation needs careful investigation, as commonly used fit statistics, such as CFI and RMSEA, yielded different qualitative conclusions in the simulated examples. We speculate that these results do not indicate model misspecification but can be attributed to small sample sizes and violations of parametric assumptions [27].

The strengths and weaknesses of PCC and CFA factor correlation complement each other. We encourage researchers to present results from both methods when assessing convergent validity during analytical validation. PCC is a conservative and stable method that demonstrates the relationship between the target and reference measures and may provide a lower bound of the true correlation. Conversely, CFA factor correlation offers an optimistic but intuitive estimate, serving as an upper bound. The true correlation likely lies between the results from these two methods.

Furthermore, multiple reference measures collected at a higher time resolution (e.g., daily instead of weekly) can increase the information available for mapping the sDHT to these reference measures, providing a more comprehensive presentation of the sDHT’s convergent validity. Using an MLR approach with daily collected reference measures notably boosted the explainable variance from the sDHT, compared to when the reference measure was only collected once.

### Novelty of the work

We operationalized the analytical validity of an sDHT using psychometric concepts (latent and observable variables). Our approach provides investigators with a general-use simulation-based framework for evaluating how well an sDHT corresponds to reported reference measures. Our approach can be adopted to other measures, beyond the physical activity case presented here.

Investigators can use our simulation code to explore various analytical validation scenarios for novel measures. The simulation code, including data generation methods, simulated data, and guidance on adapting these materials to different scenarios are publicly available on the website of the Digital Medicine Society [28] (see also the data availability statement).

We evaluated CFA factor correlation as a method for handling data believed to be generated by latent traits, alongside PCC. We believe our study is the first to adopt this approach to conduct analytical validation of sDHTs and provide a simulation toolkit for researchers to test out various scenarios *in silico* before starting data collection.

### Limitations and future research

Future research may consider specifying data generation methodologies in a manner that explicitly controls the true relationship between the sDHT and the reference measure, such as explicitly controlling the true correlation between the measures. This would allow a researcher to specify a single parameter, independent of simulation repetitions, that the statistical models can attempt to identify, thereby estimating the true bias of each model, rather than relying on empirical bias as we did here.

When modeling missingness, the MCAR mechanism was chosen to recreate missing data patterns observed in the collection of physical activity data during clinical trials [29]. The simulation revealed that whether values were MCAR or MNAR did not substantially impact the performance of the methods. Further work could explore the impact of other missing data mechanisms on the performance of statistical models, particularly mechanisms that more closely model observed real-world trends. These might encompass mechanisms applied at the epoch level instead of the summary level, such as an inverted MNAR method (i.e., making smaller data points more likely to be deleted than larger ones) [30]. Additionally, future work should investigate the impact of missing reference measure data on the analysis results, as the current simulation only included missing data for the target sDHT measure.

Our data generation approaches did not explicitly account for demographic factors. We acknowledge the importance of these factors in real-world scenarios but simulating them was beyond the scope of this work.

Finally, statistical methods examining errors-in-variables, such as Deming regression, may warrant exploration. These models would be particularly suitable because they account for error in the predictor variables. Another method that merits investigation is CFA when modeling multiple reference measures. A potentially suitable correlated two-factor model could involve loading the items from the weekly COAs and the daily assessments from the daily PRO onto separate factors, which are then loaded onto a combined “reference measure” factor, correlated against a target measure factor with each day of sDHT data loaded as separate indicators. The two-factor CFA model we used could also be modified to explore if the model fit can be improved for small sample sizes and other conditions, such as introducing covariances between consecutive days of target measure data, which more closely aligns with the DGM design.

## Supporting information

S1 File Further details on the simulation parameters and empSEs and empirical biases of correlation methods. (DOCX)

## References

[1] Goldsack JC, Coravos A, Bakker JP, Bent B, Dowling AV, Fitzer-Attas C, et al. Verification, analytical validation, and clinical validation (V3): the foundation of determining fit-for-purpose for Biometric Monitoring Technologies (BioMeTs). NPJ Digit Med. 2020;3:55. doi: 10.1038/s41746-020-0260-4 32337371 PMC7156507

[2] DiMe. V3 : An extension to the V3 framework to ensure user-centricity and scalability of sensor-based digital health technologies. https://datacc.dimesociety.org/resources/v3-an-extension-to-the-v3-framework-to-ensure-user-centricity-and-scalability-of-sensor-based-digital-health-technologies/. 2024. Accessed 2024 June 14. 10.1038/s41746-024-01322-2 PMC11760348 39856145

[3] Bakker JP, McClenahan SJ, Fromy P, Turner S, Peterson BT, Vandendriessche B, et al. A hierarchical framework for selecting reference measures for the analytical validation of sensor-based digital health technologies. JMIR Preprints. 2024. 10.2196/58956 PMC11845878 39918870

[4] Biomarkers Definitions Working Group. Biomarkers and surrogate endpoints: preferred definitions and conceptual framework. Clin Pharmacol Ther. 2001;69(3):89–95. doi: 10.1067/mcp.2001.113989 11240971

[5] Center for Drug Evaluation, Research. Surrogate Endpoint Resources for Drug and Biologic Development. https://www.fda.gov/drugs/development-resources/surrogate-endpoint-resources-drug-and-biologic-development. 2021. Accessed 2024 June 13.

[6] Wittkampf KA, Naeije L, Schene AH, Huyser J, van Weert HC. Diagnostic accuracy of the mood module of the Patient Health Questionnaire: a systematic review. Gen Hosp Psychiatry. 2007;29(5):388–95. doi: 10.1016/j.genhosppsych.2007.06.004 17888804

[7] Paulsen JS, Wang C, Duff K, Barker R, Nance M, Beglinger L. Challenges assessing clinical endpoints in early Huntington disease. Mov Disord. 2010;25(15):2595–603. 20623772 10.1002/mds.23337 PMC2978744

[8] Byrom B, McCarthy M, Schueler P, Muehlhausen W. Brain monitoring devices in neuroscience clinical research: the potential of remote monitoring using sensors, wearables, and mobile devices. Clin Pharmacol Ther. 2018;104(1):59–71. doi: 10.1002/cpt.1077 29574776 PMC6032823

[9] Bergemann T. Use of accelerometer data to evaluate physical activity as a surrogate endpoint in heart failure clinical trials. In: ASA Biopharmaceutical Section Regulatory-Industry Statistics Workshop. American Statistical Association. 2017.

[10] Sen-Gupta E, Wright DE, Caccese JW, Wright JA Jr, Jortberg E, Bhatkar V, et al. A Pivotal Study to Validate the Performance of a Novel Wearable Sensor and System for Biometric Monitoring in Clinical and Remote Environments. Digit Biomark. 2019;3(1):1–13. doi: 10.1159/000493642 32095764 PMC7015390

[11] Walls TA, Schafer JL. Models for intensive longitudinal data. Walls TA, Schafer JL, editors. Oxford University Press. 2006.

[12] Goetz CG. Movement Disorder Society-Unified Parkinson’s Disease Rating Scale (MDS-UPDRS): a new scale for the evaluation of Parkinson’s disease. Rev Neurol (Paris). 2010;166(1):1–4. doi: 10.1016/j.neurol.2009.09.001 19910010

[13] Evers LJW, Krijthe JH, Meinders MJ, Bloem BR, Heskes TM. Measuring Parkinson’s disease over time: The real-world within-subject reliability of the MDS-UPDRS. Mov Disord. 2019;34(10):1480–7. doi: 10.1002/mds.27790 31291488 PMC6851993

[14] Tucker CA, Bevans KB, Teneralli RE, Smith AW, Bowles HR, Forrest CB. Self-reported pediatric measures of physical activity, sedentary behavior, and strength impact for PROMIS: item development. Pediatr Phys Ther. 2014;26(4):385–92. doi: 10.1097/PEP.0000000000000074 25251790 PMC4176727

[15] Yao J, Tan CS, Lim N, Tan J, Chen C, Müller-Riemenschneider F. Number of daily measurements needed to estimate habitual step count levels using wrist-worn trackers and smartphones in 212,048 adults. Scientific Reports. 2021;11(1):9633. doi: 10.1038/s41598-021-88883-5 33953288 PMC8100112

[16] Hart TL, Swartz AM, Cashin SE, Strath SJ. How many days of monitoring predict physical activity and sedentary behaviour in older adults?. Int J Behav Nutr Phys Act. 2011;8:62. 21679426 10.1186/1479-5868-8-62 PMC3130631

[17] Dillon CB, Fitzgerald AP, Kearney PM, Perry IJ, Rennie KL, Kozarski R, et al. Number of days required to estimate habitual activity using wrist-worn geneactiv accelerometer: a cross-sectional study. PLoS One. 2016;11(5):e0109913. doi: 10.1371/journal.pone.0109913 27149674 PMC4858250

[18] Zhang GQ, Cui L, Mueller R, Tao S, Kim M, Rueschman M. The National Sleep Research Resource: Towards a Sleep Data Commons. J Am Med Inform Assoc. 2018;25(10):1351–8. 29860441 10.1093/jamia/ocy064 PMC6188513

[19] Griffiths P, Terluin B, Trigg A, Schuller W, Bjorner JB. A confirmatory factor analysis approach was found to accurately estimate the reliability of transition ratings. J Clin Epidemiol. 2022;141:36–45. doi: 10.1016/j.jclinepi.2021.08.029 34464687

[20] Little RJ, Rubin DB. Statistical analysis with missing data. John Wiley & Sons. 1986.

[21] RUBIN DB. Inference and missing data. Biometrika. 1976;63(3):581–92. doi: 10.1093/biomet/63.3.581

[22] Hu L, Bentler PM. Cutoff criteria for fit indexes in covariance structure analysis: conventional criteria versus new alternatives. Struct Equ Modeling. 1999;6(1):1–55.

[23] Kline R. Principles and Practice of Structural Equation Modeling. 5th. Ed. Guildford Press; 2023.

[24] Morris TP, White IR, Crowther MJ. Using simulation studies to evaluate statistical methods. Stat Med. 2019;38(11). 10.1002/sim.8086 PMC6492164 30652356

[25] Siepe BS, Bartoš F, Morris TP, Boulesteix AL, Heck DW, Pawel S. Simulation Studies for Methodological Research in Psychology: A Standardized Template for Planning, Preregistration, and Reporting. 10.31234/osf.io/ufgy6. 2023. PMC7616844 39541533

[26] Havlicek LL, Peterson NL. Robustness of the Pearson Correlation against Violations of Assumptions. Percept Mot Skills. 1976;43(3_suppl):1319–34. doi: 10.2466/pms.1976.43.3f.1319

[27] Lai K, Green SB. The problem with having two watches: assessment of Fit When RMSEA and CFI Disagree. Multivariate Behav Res. 2016;51(2–3):220–39. doi: 10.1080/00273171.2015.1134306 27014948

[28] DiMe. Validating Novel Digital Clinical Measures. https://datacc.dimesociety.org/validating-novel-digital-clinical-measures/. 2024. Accessed 2024 June 13.

[29] Cho S, Ensari I, Weng C, Kahn MG, Natarajan K. Factors Affecting the Quality of Person-Generated Wearable Device Data and Associated Challenges: Rapid Systematic Review. JMIR Mhealth Uhealth. 2021;9(3):e20738. doi: 10.2196/20738 33739294 PMC8294465

[30] Yingling LR, Mitchell V, Ayers CR, Peters-Lawrence M, Wallen GR, Brooks AT. Adherence with physical activity monitoring wearable devices in a community-based population: observations from the Washington, D.C., cardiovascular health and needs assessment. Transl Behav Med. 2017;7(4):719–30. 28097627 10.1007/s13142-016-0454-0 PMC5684058

